# Extensive Introgression among Ancestral mtDNA Lineages: Phylogenetic Relationships of the Utaka within the Lake Malawi Cichlid Flock

**DOI:** 10.1155/2012/865603

**Published:** 2012-05-10

**Authors:** Dieter Anseeuw, Bruno Nevado, Paul Busselen, Jos Snoeks, Erik Verheyen

**Affiliations:** ^1^Interdisciplinary Research Centre, K. U. Leuven Campus Kortrijk, Etienne Sabbelaan 53, 8500 Kortrijk, Belgium; ^2^KATHO, Wilgenstraat 32, 8800 Roeselare, Belgium; ^3^Vertebrate Department, Royal Belgian Institute of Natural Sciences, Vautierstraat 29, 1000 Brussels, Belgium; ^4^Evolutionary Ecology Group, University of Antwerp, Middelheimcampus G.V. 332, Groenenborgerlaan 171, 2020 Antwerp, Belgium; ^5^Zoology Department, Royal Museum for Central Africa, Leuvensesteenweg 13, 3080 Tervuren, Belgium; ^6^Laboratory of Biodiversity and Evolutionary Genomics, K. U. Leuven, Charles Deberiotstraat 32, 3000 Leuven, Belgium

## Abstract

We present a comprehensive phylogenetic analysis of the Utaka, an informal taxonomic group of cichlid species from Lake Malawi. We analyse both nuclear and mtDNA data from five Utaka species representing two (*Copadichromis* and *Mchenga*) of the three genera within Utaka. Within three of the five analysed species we find two very divergent mtDNA lineages. These lineages are widespread and occur sympatrically in conspecific individuals in different areas throughout the lake. In a broader taxonomic context including representatives of the main groups within the Lake Malawi cichlid fauna, we find that one of these lineages clusters within the non-Mbuna mtDNA clade, while the other forms a separate clade stemming from the base of the Malawian cichlid radiation. This second mtDNA lineage was only found in Utaka individuals, mostly within *Copadichromis* sp. “virginalis kajose” specimens. The nuclear genes analysed, on the other hand, did not show traces of divergence within each species. We suggest that the discrepancy between the mtDNA and the nuclear DNA signatures is best explained by a past hybridisation event by which the mtDNA of another species introgressed into the ancestral *Copadichromis* sp. “virginalis kajose” gene pool.

## 1. Introduction

The Lake Malawi cichlid fauna comprises over 800 species [1] offering a spectacular example of adaptive radiation with virtually all niches in the lake being filled by members of this family [2, 3]. With a few exceptions, all Lake Malawi cichlids form a monophyletic group as supported by mitochondrial [4–6] and nuclear ([7–9] but see [10]) markers as well as allozymes [11, 12].

The phylogenetic reconstruction of Lake Malawi cichlid fauna has recovered six main mitochondrial DNA (mtDNA) lineages [5, 6, 10, 13]. Two of these lineages correspond to the *Rhamphochromis* and *Diplotaxodon* genera. A third lineage contains the nonendemic riverine *Astatotilapia calliptera*. The remaining cichlid fauna has been traditionally divided into two groups: one containing predominantly the rock-dwelling species commonly called Mbuna and the second containing the remaining Lake Malawi cichlids. However, phylogenetic reconstructions have shown that both groups are artificial [5, 10, 13, 14]. Several *Lethrinops, Aulonocara,* and *Alticorpus* species (ecologically and morphologically typically assigned to the non-Mbuna) cluster within the Mbuna clade. Furthermore, the non-Mbuna genus *Copadichromis* has been shown to have representatives belonging to both the non-Mbuna clade, as well as to a separate lineage. The genus *Copadichromis*, together with the genus *Nyassachromis* and the newly erected genus *Mchenga, *constitute the Utaka, a species assemblage of midwater-feeding zooplanktivorous cichlid species. The phylogenetic position of this group remains unclear with Moran et al. [5] and Turner et al. [13] not recovering mtDNA monophyly within this assemblage: *M. eucinostomus *and *C. borleyi* were placed within the non-Mbuna clade, while *C. mloto* (reidentified as *Copadichromis* sp. “virginalis kajose”, J. Snoeks pers. obs.) and some other individuals of the *Copadichromis virginalis* complex seemed to represent a different, well-diverged lineage.

However only few Utaka specimens have been included in phylogenetic analyses so far. Therefore, currently available mtDNA phylogenies are inconclusive as to whether the Utaka are genetically associated with the non-Mbuna clade, whether they constitute an originally separate ancestral lineage, or whether only one or a few species or specimens cluster in a separate lineage. If specimens of a species cluster in genetically distant lineages, this may be a result of the retention of ancestral polymorphism, the existence of a cryptic species, or traces of a past hybridisation/introgression event. Support for these alternative hypotheses may be gained by using a multilocus approach (e.g., [10, 14–16]). We therefore combined mtDNA gene sequences with data from nuclear microsatellite loci. If the nuclear genetic signature is concordant with the mtDNA in subdividing a species into genetically separated units, this may point towards a cryptic species. On the other hand, if a mtDNA split within a species is not supported by the nuclear genetic data, this may be an indication of introgression of genetic material from another species, or of shared ancestral polymorphism.

Whereas the resolution of the specific interrelationships within the major clades remains problematic, the six main mtDNA clades of the Malawi cichlid flock are clearly delineated [5, 6, 13]. Shared polymorphism within taxa might result from incomplete lineage sorting, taxonomic inaccuracies, and/or hybridisation. While the other possibilities cannot be completely ruled out, there is a growing number of studies acknowledging the important role of hybridisation in the evolutionary history of adaptive radiations (e.g., [17–19]). In this study we present the most comprehensive mtDNA phylogeny of the Utaka assemblage so far. We aim at elucidating the phylogenetic position of the Utaka within the Malawian cichlid radiation and shed light on the causes for its taxonomic and molecular assignment inconsistency.

## 2. Material and Methods

### 2.1. Taxonomic Sampling

**Figure 1:**
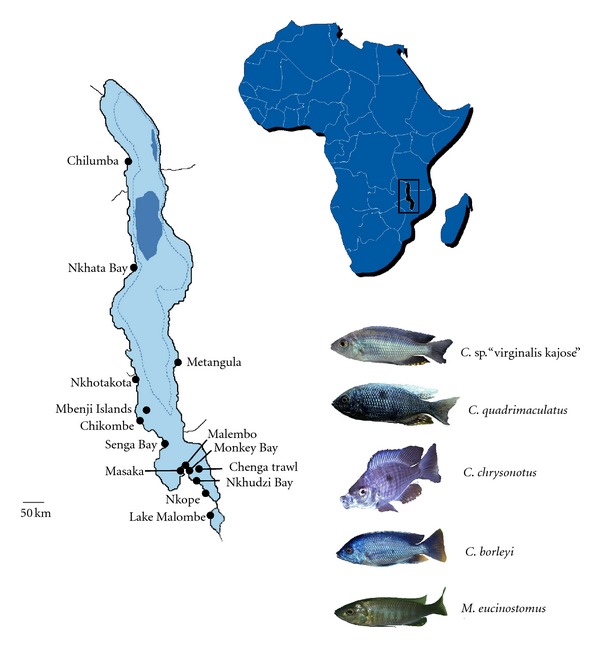
Map of Lake Malawi showing the localities sampled. A detailed listing on the origin of each specimen is presented in the Supplementary Table of the Supplementary Material available online at doi:10.1155/2012/865603.

We examined individuals of five Utaka species (*Copadichromis* sp. “virginalis kajose”, *C. quadrimaculatus, M. eucinostomus, C. chrysonotus,* and *C. borleyi*) from twelve localities throughout Lake Malawi and one locality in Lake Malombe (Figure 1). Pelvic fin clips were preserved in 100% ethanol and stored at room temperature. Voucher specimens were fixed in 10% formalin and are curated at the Royal Museum for Central Africa in Tervuren, Belgium. We included additional *Copadichromis* species that were sampled during the SADC/GEF project [1] and previously published mtDNA control region (complete D-loop) sequences of Lake Malawi cichlids, which we obtained from GenBank.

### 2.2. DNA Extraction, mtDNA Amplification, and Sequencing

Whole genomic DNA was extracted from ethanol-preserved fin clips using proteinase K digestion and salt precipitation, according to Aljanabi and Martinez [20]. DNA extracts were resuspended in 100 *μ*L of autoclaved Milli Q water. The first fragment of the mtDNA control region was sequenced for 412 Utaka specimens (179 *Copadichromis* sp. “virginalis kajose”, Genbank Accession EF211832-EF211945 and EF647210-EF647271; 55 *C. quadrimaculatus*, Genbank Accession EF647341-EF647438 and EF647578-EF647579; 67 *M. eucinostomus*, Genbank Accession EF647356-EF647390, EF647439-EF647460, EF647498-EF647505 and EF647581-EF647582; 70 *C. chrysonotus*, Genbank Accession EF647273-EF647340, and EF647571-EF647572; 41 *C. borleyi*, Genbank Accession EF647470-EF647497, EF647520-EF647531, and EF647548), using published primers by Meyer et al. [4]. We additionally sequenced the second fragment of the control region using the primers by Salzburger et al. [21] and Lee et al. [22] for 14 individuals, selected on the basis of the results of the phylogenetic reconstruction for the first fragment of the control region. Polymerase chain reactions (PCRs) were carried out in 25 *μ*L buffered reaction mixtures, containing 5 *μ*L template DNA, 5 *μ*L of each primer (2 *μ*M), 200 *μ*M of each dNTP, 2.5 *μ*L of 10x buffer (1 mM MgCl2), and 0.65 units of Red Taq Polymerase (Sigma Aldrich). PCRs were performed under the following conditions: 94°C for 120 s, followed by 35 cycles of 94°C for 60 s, 52°C for 60 s, 72°C for 120 s, followed by 72°C for 10 min. PCR products were purified following the TMQiaquick PCR purification Kit protocol and sequenced on an ABI 3130 automatic sequencer (Applied Biosystems) using standard protocols.

### 2.3. Microsatellite Variation

A total of 179 *C.* sp. “virginalis kajose”, 230 *C. chrysonotus*, 252 *C. quadrimaculatus,* and 344 *M. eucinostomus* individuals were screened for genetic variation at nine microsatellite markers: Pzeb1, Pzeb3, Pzeb4, Pzeb5 [23], UNH002 [24], TmoM5, TmoM11, TmoM27 [25], and UME003 [26]. PCRs were performed under the following conditions: 94°C for 120 s, followed by 5 cycles of 94°C for 45 s; 55°C for 45 s; 72°C for 45 s, followed by 30 cycles of 90°C for 30 s; 55°C for 30 s; 72°C for 30 s, followed by 72°C for 10 min. 10 *μ*L reaction mixes included 1 *μ*L template DNA, 0.5 *μ*M of each primer, 200 *μ*M of each dNTP, 0.26 units Taq polymerase (Sigma Aldrich, Germany), 1 *μ*L 10× reaction buffer (Sigma Aldrich). PCR amplification products were run on 6% denaturing polyacrylamide gels using an ALF Express DNA Sequencer (Amersham Pharmacia Biotech). Fragment sizes were scored with ALFWin Fragment Analyser v1.0 (Amersham Pharmacia Biotech), using M13mp8 DNA standards as external references, following van Oppen et al. [23].

### 2.4. Phylogenetic Reconstructions

For the reconstruction of the phylogenetic relationships of the Utaka, two datasets were analysed. Both were aligned using CLUSTALW [27] and visually checked afterwards using the program SEAVIEW [28]. The first dataset contained 412 *Copadichromis* spp. and *Mchenga* sp. sequences of the first fragment of the control region (328 bp). The program COLLAPSE v1.2 [29] was used to reduce this dataset to one individual sequence per haplotype for further analyses. GTR+G+I model was the best-fitted model of sequence evolution inferred by MODELTEST v3.7 [30] according to the Akaike Information Criterion (AIC). A maximum likelihood (ML) heuristic search was performed with PHYML [31] starting from a neighbour-joining (NJ) tree. Parameters of the tree and of the substitution model were optimised sequentially until no increase in likelihood was found. The program TCS [32] was used to generate a haplotype network using statistical parsimony. Based on the results of the short control region phylogenetic reconstruction, we performed a second, more computationally intensive phylogenetic analysis with a smaller dataset containing 47 representatives of the different main lineages in the Lake Malawi cichlid flock (both new sequences and sequences extracted from GenBank) to test the interrelationships between these main lineages. Sites that could not be unambiguously aligned were removed prior to analysis. The final dataset, 837 bp long, was first run through MODELTEST, which selected the TrN+I+G model (AIC criterion).

Phylogenetic inferences were carried out using maximum-parsimony (MP, 100 replicates starting from random stepwise addition trees; TBR branch swapping) with different transition-transversion weights (1 : 1, 2 : 1 and 3 : 1) in PAUP* v4.0 [27]. ML reconstructions (100 replicates starting from random stepwise addition trees; TBR branch swapping) were run in PAUP*. Sequential searches were performed by reestimating the substitution model parameters upon the best tree found and then running a new search with these parameters. This was done until no change in the likelihood of the tree or in the estimated parameters was found. Support for the internal branches in the ML tree was assessed by analysing 100 bootstrapped replicates in the program PHYML. For Bayesian inference (BI) analyses, the GTR+G+I model was used since the TrN+G+I is not implemented in MRBAYES v.3.1 [33]. Markov Chain Monte Carlo samplings were run for 25 million generations. Two runs with four chains for each run were sampled every thousandth generation until the average standard deviation of split frequencies between runs reached ~0.003. Inspection of plot of likelihood versus generation revealed that the runs had reached stability and so did the analysis of the Potential Scale Reduction Factors.

Using the Shimodaira-Hasegawa test [34], as implemented in PAUP*, we tested the relative fit of two alternative tree topologies: the forced monophyly of all Utaka specimens was compared to the best, unconstrained tree. Significance of the difference in log-likelihood between the two trees was assessed by means of the Resample Estimated Log-Likelihood test (RELL).

### 2.5. Microsatellite Data Analysis

Linkage disequilibrium between loci was tested using exact tests as implemented by GENEPOP 3.3 [35]. We estimated the number of populations present in our microsatellite dataset using the program STRUCTURE [36, 37]. We calculated the posterior probability for different numbers of putative populations (K from 1 to 18 populations) using a model-based assignment. Burn-in was set at 100,000 steps followed by 300,000 MCMC iterations at each *K*. Simulations were run five times for each *K* to check for convergence of the MCMC. We performed clustering both under the admixture model without prior population information and with correlated allele frequencies between populations. To determine the most likely number of clusters, the rate of change in the log probability of data and in the statistic Δ*K* [38] between successive *K* values was estimated using StructureHarvester [39].

## 3. Results

### 3.1. Phylogenetic Reconstructions

**Figure 2:**
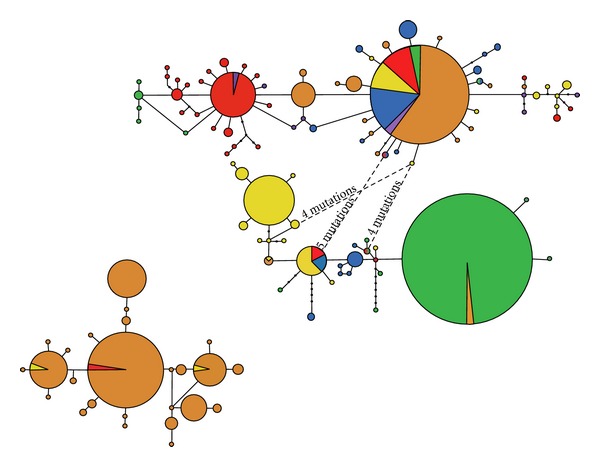
Haplotype networks of the Utaka obtained in this study. The upper network contains the majority of the specimens analysed and is separated from the lower network (named virginalis clade in text) by more than seven mutations. Each circle represents a haplotype and is coloured according to the respective species: blue: *C. borleyi*; orange: *C.* sp. “virginalis kajose”; red: *C. quadrimaculatus*; green: *C. chrysonotus* yellow: *M. eucinostomus*; purple: *C. mloto* and *C.* sp. “meta”. Size of the circles is proportional to the frequency of each haplotype as indicated in the scaled circles. Small black circles in branches represent missing haplotypes. Dashed lines represent alternative connections with the number of missing haplotypes written above the lines.

The purpose of our phylogenetic analyses was twofold. First, we assessed the phylogenetic relationships among as many specimens as available from the five Utaka species that we collected throughout the lake. For this extensive dataset, we sequenced the short (328 bp) but most variable part of the mtDNA control region. By this analysis we aimed to detect specific or geographical patterns among the Utaka species studied. Second, we attempted to resolve the phylogenetic position of the Utaka species within the Lake Malawi cichlid flock. For this purpose we sequenced the complete mitochondrial control region for representative specimens (*n* = 14) of the previous dataset and included published sequences from species representing the main lineages in the Malawian cichlid flock. A total of 115 haplotypes were found amongst the 412 Utaka short mtDNA control region sequences (Figure 2). The ML tree presented two divergent clades within the Utaka: a large clade containing circa 70% of all sequences, and a smaller group. The latter almost exclusively contained *C.* sp. “virginalis kajose” individuals (125 *C.* sp. “virginalis kajose” individuals out of 179 sequenced clustered within this clade), together with two (out of 67) *M. eucinostomus* and one (out of 55) *C. quadrimaculatus* individuals. Both mtDNA clades were present lake-wide in nearly all localities sampled, and within each lineage distinct geographic structuring was absent.

**Figure 3:**
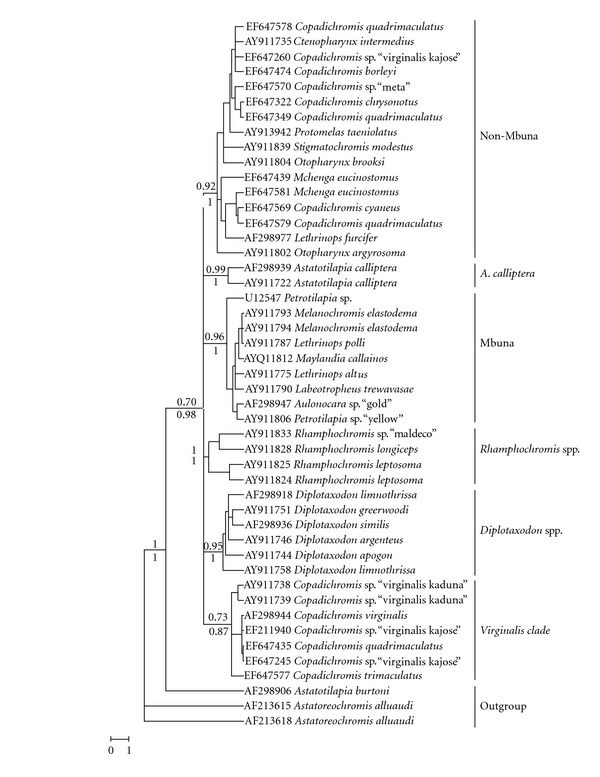
Maximum-likelihood reconstruction (PhyML) of the Lake Malawi cichlid flock using the complete mtDNA control region. Numbers next to the branches show bootstrap percentage support (upper) and bayesian posterior probabilities (below) of the main clades. Clade names follow denomination given in text. *Astatoreochromis alluaudi* and *Astatotilapia burtoni* represent the outgroups. Scale bar indicates substitutions per nucleotide site.

The complete mtDNA control region phylogenetic reconstructions using MP (with the different weighting schemes), ML, and BI consistently recovered the 6 main clades among the Malawian cichlids (Figure 3): (i) a lineage containing most non-Mbuna and Utaka species (non-Mbuna clade hereafter); (ii) a clade containing only *Copadichromis* individuals (virginalis clade hereafter); (iii) a Mbuna clade containing all Mbuna species plus some deep-water *Lethrinops* species and an *Aulonocara* specimen; (iv) a *Diplotaxodon* clade; (v) a *Rhamphochromis* clade; (vi) a clade containing *A. calliptera*. For these clades, bootstrap values and posterior probabilities displayed high node support values, except for the virginalis clade, which had a bootstrap support of 73 and a posterior probability of 0.87 (Figure 3). The phylogenetic relationships between the clades remained, however, unresolved: their branching order was variable, depending on the reconstruction methods used and was even resolved as a polytomy in the Bayesian analysis. The Shimodaira-Hasegawa test indicated a significant difference in likelihood score between the two topologies examined (*P* = 0.03), giving preference to the unconstrained topology (where Utaka are paraphyletic) over the best tree obeying to the monophyly of all Utaka specimens analysed.

### 3.2. Microsatellites

**Figure 4:**
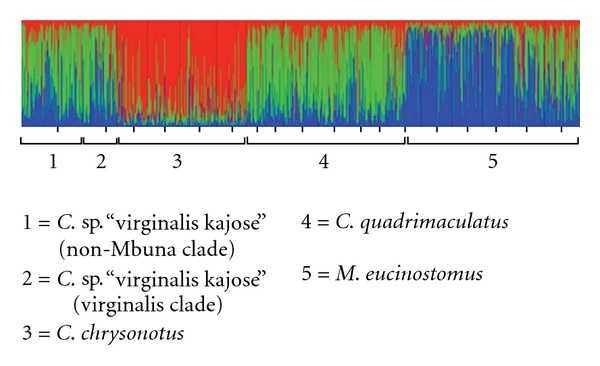
Bar plot result of the STRUCTURE assignment test for *K* = 3 under the nonadmixture model. The two mtDNA groups within *C.* sp. “virginalis kajose” (non-Mbuna and virginalis clade) cannot be distinguished from each other based on the nuclear markers, whereas *C. chrysonotus* and *M. eucinostomus* clearly differentiate although they share the same mtDNA haplotype lineage.

Linkage disequilibrium tests across loci and populations revealed no significant allelic associations. The model-based clustering approach implemented in the program STRUCTURE yielded estimated Ln probabilities for 1 ≤ *K* ≤ 18 ranging from –37678 to –34160 with the highest posterior probability and Δ*K*  (=20.605) for *K* = 3. In the most likely scenario, STRUCTURE assigned *M. eucinostomus* and *C. chrysonotus* to two different groups, while *C. quadrimaculatus* and *C. *sp. “virginalis kajose” formed a third cluster (Figure 4).

## 4. Discussion

The phylogenetic inferences of the Utaka assemblage performed herein showed that it contains two genetically distant and geographically widespread mtDNA lineages. The two lineages have been observed before [5, 10, 13, 14] but this is the first study to reveal the paraphyly not only of the genus *Copadichromis* in individuals from throughout Lake Malawi, but also of three (of the five analysed) Utaka species (based on the short mtDNA sequences). In a wider taxonomic context involving the other Malawian cichlid lineages, the most abundant of the two lineages in the Utaka clustered within the non-Mbuna mtDNA clade, while the other formed a separate clade containing exclusively Utaka specimens, mostly *C.* sp. “virginalis kajose” individuals. The paraphyly of the Utaka does not represent an artefact in our analyses, as corroborated by the long and well-supported branches that connect the non-Mbuna and the virginalis clades, as well as by the significant result of the Shimodaira-Hasegawa test.

One possible explanation is that the Utaka share ancestral polymorphic alleles and/or represent a truly paraphyletic group containing multiple lineages that have undergone convergent evolution. Importantly, the occurrence of two divergent mtDNA lineages within the Utaka is related neither to taxonomic clustering, nor to geographical structuring. If the two haplogroups observed within the Utaka indeed correspond to two ancestral lineages that are genetically isolated for such a long time that their mtDNA genotypes have become so deeply diverged, we would expect this to be also reflected in the nuclear genome of the species. However, we did not find any subdivision of nuclear gene pools that corresponds to the deep mtDNA divergence, neither across the Utaka species, nor within *C.* sp. “virginalis kajose” which yields the majority of the individuals in the divergent virginalis clade as well as a large number of individuals in the non-Mbuna clade. It thus seems unlikely that the presence of a cryptic species is the cause of the mtDNA divergence within *C.* sp. “virginalis kajose.” Recently published phylogenetic reconstructions using AFLP loci [10, 14] also showed a discordance between the nuclear and mitochondrial placement of *Copadichromis virginalis *within the Malawi cichlid radiation, supporting our finding that the observed paraphyly of the Utaka and of *C. *sp. “virginalis kajose” is unlikely to be the result of incomplete ancestral lineage sorting or true paraphyly.

Alternatively, a disparate pattern of divergence between mitochondrial and nuclear DNA among conspecific individuals may be the result of a past hybridisation and introgression event, a process which has been documented in Malawian cichlids before (e.g., [40–42]) and for which evidence is accumulating (e.g., [10, 14, 16, 43]). Under this hypothesis we advance two possibilities regarding the original position of the Utaka within the Malawi cichlid phylogeny. A first scenario assumes that all Utaka species formerly constituted a separate ancestral clade within the Malawi cichlid flock, corresponding with the current virginalis clade. Subsequent unidirectional introgression of mtDNA from non-Mbuna into the Utaka could then explain the observed clustering of Utaka specimens within the non-Mbuna lineage. This scenario would involve that either all, or the ancestors of the current Utaka species, would have been extensively hybridised with a non-Mbuna species, resulting in the almost complete replacement of the original mtDNA of the Utaka. A second scenario assumes that all Utaka species initially belonged to the non-Mbuna lineage and a species from a distant mtDNA lineage hybridised with *Copadichromis* species. The mtDNA detected in the virginalis clade may then represent the introgressed mtDNA.

Interestingly and despite our extensive taxonomic sampling, the maternal species involved in the putative hybridisation event remains unidentified as the virginalis clade only contained representatives of the Utaka assemblage. It would seem that the species with which *Copadichromis* spp. hybridised either has thus far not been subjected to molecular studies or may no longer be present in the lake. Empirical evidence for or against the above scenarios can be gained by examining mtDNA of supplementary Utaka species to validate whether the majority of the taxa cluster is within the non-Mbuna clade or within the virginalis clade. The more Utaka species cluster within the non-Mbuna clade, the less probable becomes the first scenario. Regardless of which of the two mtDNA lineages is the original or the introgressing one, and irrespective of the maternal species involved in the hybridisation event, our results show that the two mtDNA lineages have persisted within the gene pool of *Copadichromis* sp. “virginalis kajose” for a rather long period, as suggested by the diversity displayed by either of these two lineages (Figure 2). It thus suggests that either the population size of this species has remained very high since the hybridisation event (such that genetic drift would represent a lesser issue) or that some other mechanism is maintaining the two lineages within the same species (e.g., balancing or frequency-dependent selection).

Interspecific gene flow is increasingly recognized as an important factor in shaping speciation (e.g., [17–19, 44, 45]). Progressively more examples for hybridization are known from African cichlid fish: among Lake Tanganyika's cichlids evidence is found for ancient introgression (e.g., [15, 46–48]) and a complete replacement [49] of mtDNA in multiple tribes of the cichlid assemblage. From Lake Malawi, evidence for deep introgression leaving a long-term signal in its haplochromine radiation [10, 14, 43], as well as evidence for more recent natural hybridisation [16, 50, 51] among Malawi cichlids, has been provided. In the Lake Victoria cichlid flock recent or ongoing hybridisation [52–54] presumably affects large parts of the species' genomes by homogenization [54, 55], hampering the reconstruction of its young evolutionary history [54, 56, 57], yet potentially seeding the process of speciation [58] but see [55]. In Cameroonian crater lakes the hybridisation of two ancient lineages resulted in the formation of a new and ecologically highly distinct species [59]. Also for *Steatocranus *cichlids from the Congo basin it was recently shown that ancient as well as recent introgression of genes and hybridisation produced a genomic network that potentially promoted divergence and speciation [60]. Our results chime well with previous studies reporting hybridisation in the early stages of a cichlid radiation. Our findings reconcile with the recently reported evidence for ancient introgression between Mbuna and deep-benthic cichlids at the base of the Malawi radiation [14]. Remarkably, in our study we could not separate *Copadichromis *sp. “virginalis kajose” and *C. quadrimaculatus*, two phenotypically distinct taxa, by our microsatellite markers. However, it has already been reported that the performance of a clustering method may become poor for *Fst*'s below 0.05 ([61], J. Pritchard, *pers. comm.*). The estimates of population differentiation were low in both species (*θ* = 0.006 in *C. *sp. “virginalis kajose” and *θ* = 0.007 in *C. quadrimaculatus*, reported in [62]), and slightly higher among the two species (*θ* = 0.01). Whether this observation might yield a demonstration of the relative ease of hybridisation among phenotypically well-differentiated taxa [14, 43] or be the result of an insufficient resolution of the markers used, deserves further research.

## Supplementary Material

List of the specimens, their sampling location, and the mtDNA clade they were assigned to in this study. Clade names follow denomination given in the text.
