# Supplementary material for: Extensive Introgression among Ancestral mtDNA Lineages: Phylogenetic Relationships of the Utaka within the Lake Malawi Cichlid Flock
Source: Int J Evol Biol. 2012 May 10;2012:865603. doi: 10.1155/2012/865603 (PMC3357950; doi:10.1155/2012/865603)
Supplement: Supplementary file 1 — List of the specimens, their sampling location, and the mtDNA clade they were assigned to in this study. Clade names follow denomination given in the text. [file 865603.f1.pdf]

Supplementary Table: List of the specimens, their sampling location, and the mtDNA clade they were assigned to in this study. Clade names follow denomination given in the text.

NCBI Accession	Species	Sampling location	mtDNA clade
EF211836<br>EF211839<br>EF211887<br>EF211889<br>EF211893-EF211895<br>EF211897-EF211899<br>EF211902	*Copadichromis* sp. 'virginalis kajose'	Senga Bay	non-Mbuna
EF211851<br>EF211856<br>EF211913	*Copadichromis* sp. 'virginalis kajose'	Chilumba	non-Mbuna
EF211870-EF211871<br>EF211876- EF211879<br>EF211881-EF211882<br>EF211928<br>EF211930- EF211935<br>EF211938<br>EF211942<br>EF211944	*Copadichromis* sp. 'virginalis kajose'	Nkhudzi Bay	non-Mbuna
EF647211<br>EF647216<br>EF647219<br>EF647222<br>EF647226	*Copadichromis* sp. 'virginalis kajose'	Lake Malombe	non-Mbuna
EF647230- EF647231<br>EF647233-EF647239	*Copadichromis* sp. 'virginalis kajose'	Chenga trawler	non-Mbuna
EF647248	*Copadichromis* sp. 'virginalis kajose'	Nkhotakota	non-Mbuna
EF647251<br>EF647253-EF647260	*Copadichromis* sp. 'virginalis kajose'	Nkhata Bay (Sanga Beach)	non-Mbuna
EF647261-EF647272	*Copadichromis* sp. 'virginalis kajose'	Mbenji Islands	non-Mbuna
EF647273-EF647285	*Copadichromis chrysonotus*	Lake Malombe	non-Mbuna
EF647286-EF647297	*Copadichromis chrysonotus*	Chilumba	non-Mbuna
EF647298-EF647309	*Copadichromis chrysonotus*	Monkey Bay (Mwawa Beach)	non-Mbuna
EF647310-EF647312<br>EF647332-EF647340	*Copadichromis chrysonotus*	Masaka	non-Mbuna
EF647313-EF647319	*Copadichromis chrysonotus*	Senga Bay	non-Mbuna

EF647320-EF647331	*Copadichromis chrysonotus*	Nkhotakota	non-Mbuna
EF647341-EF647343<br>EF647391-EF647398	*Copadichromis quadrimaculatus*	Chilumba	non-Mbuna
EF647344-EF647355	*Copadichromis quadrimaculatus*	Mbenji Islands	non-Mbuna
EF647356- EF647359<br>EF647361-EF647367	*Mchenga eucinostomus*	Chilumba	non-Mbuna
EF647368-EF647379	*Mchenga eucinostomus*	Nkope	non-Mbuna
EF647381-EF647390	*Mchenga eucinostomus*	Nkhotakota	non-Mbuna
EF647399-EF647411	*Copadichromis quadrimaculatus*	Nkhotakota	non-Mbuna
EF647412-EF647419	*Copadichromis quadrimaculatus*	Nkhata Bay	non-Mbuna
EF647430- EF647434<br>EF647436-EF647438	*Copadichromis quadrimaculatus*	Malembo	non-Mbuna
EF647439-EF647450	*Mchenga eucinostomus*	Senga Bay	non-Mbuna
EF647451-EF647460	*Mchenga eucinostomus*	Chikombe	non-Mbuna
EF647470-EF647481	*Copadichromis borleyi* 'Kadango'	Wild caught Makanjila Point, Stuart Grant	non-Mbuna
EF647482-EF647497	*Copadichromis borleyi* 'Eastern'	Wild caught Makanjila Point, Stuart Grant	non-Mbuna
EF647498-EF647505	*Mchenga eucinostomus*	Nkhata Bay	non-Mbuna
EF647518-EF647519	*Copadichromis trimaculatus*	Chikombe	non-Mbuna
EF647520-EF647531	*Copadichromis borleyi*	Chilumba	non-Mbuna
EF647570	*Copadichromis* sp. 'meta'	Metangula	non-Mbuna
EF647569	*Copadichromis cyaneus*	Tchulutcha Reef in front of Metangula	non-Mbuna
EF647577	*Copadichromis trimaculatus*	Iwela, 10 miles N of Manda Bay	virginalis clade
EF647209-EF647210<br>EF647212-EF647215<br>EF647217-EF647218<br>EF647220-EF647221<br>EF647223-EF647225<br>EF647228-EF647229	*Copadichromis* sp. 'virginalis kajose'	Lake Malombe	virginalis clade
EF211848-EF211850<br>EF211852-EF211855<br>EF211857-EF211867<br>EF211905-EF211912<br>EF211914- EF211924	*Copadichromis* sp. 'virginalis kajose'	Chilumba	virginalis clade
EF647252	*Copadichromis* sp. 'virginalis kajose'	Nkhata Bay	virginalis clade
EF647240-EF647247<br>EF647249-EF647250	*Copadichromis* sp. 'virginalis kajose'	Nkhotakota	virginalis clade

EF647261-EF647272	*Copadichromis* sp. 'virginalis kajose'	Mbenji Islands	virginalis clade
EF211832-EF211835<br>EF211837-EF211838<br>EF211840-EF211847<br>EF211888<br>EF211890-EF211892<br>EF211896<br>EF211900-EF211901<br>EF211903-EF211904	*Copadichromis* sp. 'virginalis kajose'	Senga Bay	virginalis clade
EF211868-EF211869<br>EF211872-EF211875<br>EF211880<br>EF211883-EF211886<br>EF211925-EF211927<br>EF211929<br>EF211934<br>EF211936-EF211937<br>EF211939-EF211941<br>EF211943<br>EF211945-EF211946	*Copadichromis* sp. 'virginalis kajose'	Nkhudzi Bay	virginalis clade
EF647232	*Copadichromis* sp. 'virginalis kajose'	Chenga trawl	virginalis clade
EF647360	*Mchenga eucinostomus*	Chilumba	virginalis clade
EF647380	*Mchenga eucinostomus*	Nkhotakota	virginalis clade
EF647435	*Copadichromis quadrimaculatus*	Malembo	virginalis clade
